# Supplementary material for: Comparison of the Hemocompatibility of an Axial and a Centrifugal Left Ventricular Assist Device in an In Vitro Test Circuit
Source: J Clin Med. 2022 Jun 15;11(12):3431. doi: 10.3390/jcm11123431 (PMC9225365; doi:10.3390/jcm11123431)
Supplement: Supplementary file 1 [file jcm-11-03431-s001.zip › jcm-1705532-SI.pdf]

Supplementary Figure S1

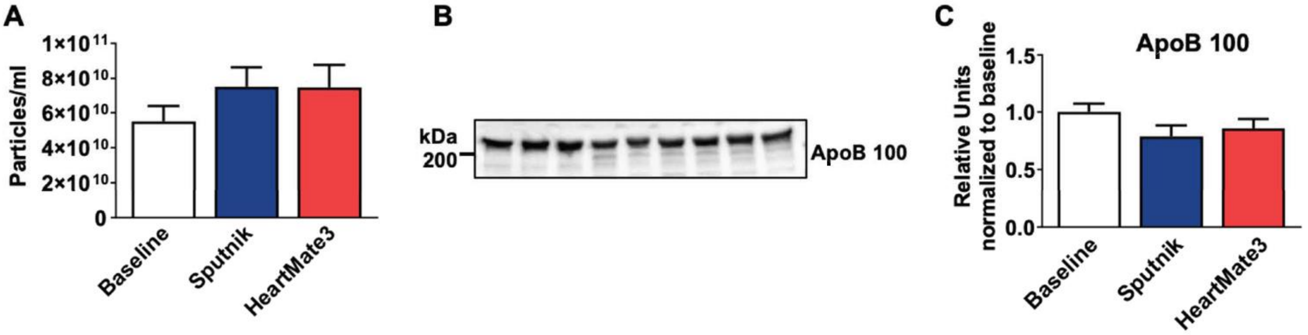

Figure S1. Quantification of EVs and Analysis of ApoB 100. **(A)** Quantification of EVs by Nanoparticle tracking analysis **(B)** Representative Western Blot Images of ApoB 100 in EVs isolated at baseline and after 6 h of circulation using the Sputnik and HeartMate 3 pump. **(C)** Western blot bands were quantified and normalized to the corresponding total protein amount visualized in the acrylamide gels by stain-free technology and then compared to baseline. Data represent means ± SEM of six independent samples.

Nanoparticle tracking analysis (Fig. S1 A) showed only a slight and statistically not significant increase of EVs from $5.50x10^{10}$±$8.83x10^{09}$ particles/ml in the baseline sample to $7.49x10^{10}$±$1.13x10^{10}$ particles/ml in the Sputnik and $7.45x10^{10}$±$1.32x10^{10}$ particles/ml in the HeartMate 3 group, respectively.
The non-EV protein ApoB, which is the primary apolipoprotein of chylomicrons, VLDL, and LDL particles, could be detected in all samples, before and after circulation (Fig. S1 B-C). This indicates that the precipitation protocol we used to isolate EVs in this study coisolated lipoproteins and that the amount of contaminating particles does not increase during circulation.

We could successfully isolate EVs by precipitation from baseline samples and after 6 h of circulation. Although this method of purification is controversially discussed in the literature [1,2], as it coprecipitates contaminating lipoproteins, it is still one of the best ways to isolate EVs from very low sample volumes. Other methods require specialized equipment (e.g., ultracentrifugation), long hands-on time and user training, which can result in low reproducibility. With the ExoQuick Ultra kit, we were able to isolate EVs from only 200 µl plasma in an adequate time frame. EVs were visualized by TEM and, in the baseline samples, typical EVs were detected with a size range around 100 nm. After circulation, EVs could hardly be detected by TEM due to hemolysis and the resulting excessive cell debris. EVs were quantified by NTA, and a slight but insignificant increase was detected in the Sputnik and HeartMate 3 circuits. However, when we performed Western blot analysis of our isolated EVs, we detected a significant increase in two common EV markers: Flotillin-1 and CD9 [3], which were both increased in the Sputnik EVs. The difference between the NTA quantification and Western blot results could be explained by the lack of purity. We also probed our membranes for apolipoprotein B, which is the main component of chylomicrons, VLDL and LDL particles [4]. Apolipoprotein B could be detected in all samples, independent of the LVAD. Therefore, contaminating vesicles were co-isolated with our EVs, which resulted in a high, unspecific background, since NTA cannot discriminate between lipoproteins and EVs [5]. The Western blot images, on the other hand, reflect true vesicular markers and show a drastic increase in EVs after LVAD circulation.

References

1. Veerman, R.E.; Teeuwen, L.; Czarnewski, P.; Güclüler Akpinar, G.; Sandberg, A.; Cao, X.; Pernemalm, M.; Orre, L.M.; Gabrielsson, S.; Eldh, M. Molecular Evaluation of Five Different Isolation Methods for Extracellular Vesicles Reveals Different Clinical Applicability and Subcellular Origin. *J. Extracell. Vesicles* **2021**, *10*, e12128, doi:10.1002/jev2.12128.

2. Brennan, K.; Martin, K.; FitzGerald, S.P.; O'Sullivan, J.; Wu, Y.; Blanco, A.; Richardson, C.; Mc Gee, M.M. A Comparison of Methods for the Isolation and Separation of Extracellular Vesicles from Protein and Lipid Particles in Human Serum. *Sci. Rep.* **2020**, *10*, 1039, doi:10.1038/s41598-020-57497-7.

3. Kowal, J.; Arras, G.; Colombo, M.; Jouve, M.; Morath, J.P.; Primdal-Bengtson, B.; Dingli, F.; Loew, D.; Tkach, M.; Théry, C. Proteomic Comparison Defines Novel Markers to Characterize Heterogeneous Populations of Extracellular Vesicle Subtypes. *Proc. Natl. Acad. Sci. U. S. A.* **2016**, *113*, E968-977, doi:10.1073/pnas.1521230113.
4. Sódar, B.W.; Kittel, Á.; Pálóczi, K.; Vukman, K.V.; Osteikoetxea, X.; Szabó-Taylor, K.; Németh, A.; Sperlágh, B.; Baranyai, T.; Giricz, Z.; et al. Low-Density Lipoprotein Mimics Blood Plasma-Derived Exosomes and Microvesicles during Isolation and Detection. *Sci. Rep.* **2016**, *6*, 24316, doi:10.1038/srep24316.
5. Comfort, N.; Cai, K.; Bloomquist, T.R.; Strait, M.D.; Ferrante, A.W.; Baccarelli, A.A. Nanoparticle Tracking Analysis for the Quantification and Size Determination of Extracellular Vesicles. *J. Vis. Exp. JoVE* **2021**, doi:10.3791/62447.
